# The MOC31PE immunotoxin reduces cell migration and induces gene expression and cell death in ovarian cancer cells

**DOI:** 10.1186/1757-2215-7-23

**Published:** 2014-02-15

**Authors:** Merete Thune Wiiger, Hemaseh Bideli, Øystein Fodstad, Kjersti Flatmark, Yvonne Andersson

**Affiliations:** 1 Department of Tumor Biology, Institute for Cancer Research, Norwegian Radium Hospital, Oslo University Hospital, Oslo, Norway; 2 Department of Gastroenterological Surgery, Norwegian Radium Hospital, Oslo University Hospital, University of Oslo, Oslo, Norway; 3 University of Oslo, Oslo, Norway

**Keywords:** Immunotoxin, EpCAM, Ovarian cancer, Gene-expression, NR4A3, MOC31

## Abstract

**Background:**

The standard treatment of ovarian cancer with chemotherapy often leads to drug resistance and relapse of the disease, and the need for development of novel therapy alternatives is obvious. The MOC31PE immunotoxin binds to the cell surface antigen EpCAM, which is expressed by the majority of epithelial cancers including ovarian carcinomas, and we studied the cytotoxic effects of MOC31PE in ovarian cancer cells.

**Methods:**

Investigation of the effects of MOC31PE treatment on protein synthesis, cell viability, proliferation and gene expression of the ovarian cancer cell lines B76 and HOC7.

**Results:**

MOC31PE treatment for 24 h caused a dose-dependent reduction of protein synthesis with ID_50_ values of less than 10 ng/ml, followed by reduced cell viability. In a gene expression array monitoring the expression of 84 key genes in cancer pathways, 13 of the genes were differentially expressed by MOC31PE treatment in comparison to untreated cells. By combining MOC31PE and the immune suppressor cyclosporin A (CsA) the MOC31PE effect on protein synthesis inhibition and cell viability increased tenfold. Cell migration was also reduced, both in the individual MOC31PE and CsA treatment, but even more when combining MOC31PE and CsA. In tumor metastasis PCR arrays, 23 of 84 genes were differentially expressed comparing CsA versus MOC31PE + CsA treatment. Increased expression of the tumor suppressor KISS1 and the nuclear receptor NR4A3 was observed, and the differential candidate gene expression was confirmed in complementary qPCR analyses. For NR4A3 this was not accompanied by increased protein expression. However, a subcellular fractionation assay revealed increased mitochondrial NR4A3 in MOC31PE treated cells, suggesting a role for this protein in MOC31PE-induced apoptotic cell death.

**Conclusion:**

The present study demonstrates that MOC31PE may become a new targeted therapy for ovarian cancer and that the MOC31PE anti-cancer effect is potentiated by CsA.

## Background

Ovarian cancer is the leading cause of death from gynecological cancers and the patients are commonly diagnosed late with advanced disease. In general, the patients respond well to the primary treatment involving cytoreductive surgery and chemotherapy. However, more than 70% of the patients relapse, and in the recurrent disease, resistance to chemotherapeutic drugs is common [1,2]*.* New targeted therapies are under evaluation, and immunotoxins (ITs) may represent an interesting alternative. ITs consist of an antibody, that with high affinity binds to the target antigen on the cancer cell surface, and a covalently bound toxin. Our MOC31PE immunotoxin binds to the cell surface antigen EpCAM, which is expressed by the majority of epithelial cancers including ovarian carcinomas. Upon internalisation *Pseudomonas* exotoxin A (PE) inhibits protein synthesis by ADP-ribosylation of elongation factor 2 and induces apoptosis. EpCAM is a transmembrane glycoprotein, functioning as an epithelial-specific cell-cell adhesion molecule and may be involved in cellular signaling, migration, proliferation, and differentiation [3]. Recently, it has been suggested that EpCAM is a cancer stem cell marker and may be expressed by cells undergoing epithelial to mesenchymal transition (EMT), lacking other epithelial markers [4]. EMT-like cellular processes may be important during cancer metastasis, and EpCAM is thus an excellent candidate for therapeutic targeting of epithelial cancers. In a retrospective study of 500 ovarian cancer patients, EpCAM showed consistently high expression across different tumor stages and subtypes [5] and the protein was over-expressed in cancerous tissues compared with non-cancerous ovarian surface epithelium and inclusion cysts [6]. Notably, MOC31PE also induces cell death in chemotherapy-resistant cancer cells [7] and may hence be used in patients with recurrent disease lacking other therapeutic options.

The immune suppressor cyclosporin A (CsA) was introduced in combination with IT to inhibit the host immune response during repeated IT administrations. In parallel with reduced anti-IT antibody production, synergistic cytotoxic effects were observed *in vitro* and *in vivo*[8]. The immunosuppressive effect of CsA is caused by binding to cyclophilin A (CypA) [9]. This complex binds and inhibits calcineurin a key enzyme for IL-2 production in T-cells. CypA over-expression has been reported in many human cancers and has also been suggested as a potential therapeutic target [10]. Interestingly, CsA has been reported to reverse chemotherapeutic resistance in patients with recurrent ovarian cancer [11,12]. In the present work, we have studied the effects of MOC31PE treatment alone and in combination with CsA on protein synthesis, cell proliferation, viability, and migration on the ovarian cancer cell lines B76 [13] and HOC7 [14], which both express high amounts of EpCAM. Furthermore, MOC31PE-induced alterations in gene transcription were quantified in two different PCR-arrays: Cancer Pathway Finder and Tumor Metastasis.

## Materials and methods

### Materials

RPMI-1640, PBS, Glutamax, and Hepes were obtained from Lonza (Austria). Fetal calf serum was purchased from PAA (GE Healthcare, UK), MEM w/o leucine, 0.25% Trypsin/EDTA from Gibco, and YoYo-1 fluorescent dsDNA staining from Molecular Probes (Life Technologies, UK), and tritiated Leucine from Perkin Elmer (Waltham, MA). Cyclosporine A was purchased from Calbiochem (San Diego, CA) and dissolved in ethanol to 8.3 mM stock solution. The GenElute Mammalian total RNA kit and general laboratory chemicals were from Sigma Aldrich (St. Louis, MO), the Cell Titer 96 AqueousOne solution (MTS) cell proliferation assay was from Promega (Madison, WI). RT^2^ Profiler PCR Array System, including the cDNA synthesis kit, and SYBR green were from SABiosciences (Qiagen Nordic). Chemicals for validation of gene expression were from Applied (Life Technologies, UK). Plastic ware for cell culture was from Nunc (Thermo Scientific), gels and buffers for protein electrophoresis from Life Technologies, HRP-conjugated antibodies from Dako (DK), and chemiluminescent super-signal substrate from Thermo Scientific.

### Cells and immunotoxin

The human ovarian cancer cell lines B76 [13] and HOC-7 [14] were a gift from Dr C. Marth (Innsbruck Medical University, Innsbruck, Austria). In this study B76 was our main cell line and HOC-7 was used to confirm key results. The cell lines were cultivated in RPMI 1640 medium added Glutamax, Hepes and 8% heat-inactivated fetal calf serum. The monoclonal antibody MOC31 [15] binds epithelial cell adhesion molecule (EpCAM, CD326) and was conjugated to whole *Pseudomonas* exotoxin A as previously described [16].

### Protein synthesis and cell viability

The [^3^H]-leucine incorporation assay was used to quantify protein synthesis [16] and the Cell Titer 96 AqueousOne solution (MTS) assay was used to determine cell viability as previously described [17].

### Cell proliferation, membrane damage and scratch-wound healing in the IncuCyte

Cells were seeded in 96 well plates and grown to ≈ 50% confluency, transferred to the IncuCyte (Essen BioSciences, Ann Arbor, Mi) after the medium was replaced with fresh medium with or without IT and/or CsA. Membrane damage was measured after adding YoYo-1, a dye that emit fluorescence when it binds to double-stranded DNA. The cytotoxic index is defined as the number of fluorescent objects in a well, divided by the total number of fluorescent objects obtained after 0.1% Triton X-100 is added to open all cells in the well. For migration studies, the wound maker tool was used to make scratch wounds in confluent cell culture monolayers in 96 well image-lock plates (Essen BioSciences). Plates were incubated in the IncuCyte for 24 h and an integrated metric called relative wound density (RWD) was used to quantify effects on migration. This metric measures the cell density in the wound area relative to the cell density outside the wound area.

### RNA isolation and PCR array analyses

The cells were seeded in 6 well plates, grown to ≈ 80% confluency and treated for 24 h before RNA was isolated from adherent cells using the GenElute Mammalian total RNA kit (Sigma Aldrich) and quantified in a Picodrop spectrophotometer (Picodrop Ltd, UK). RNA isolated for PCR array assays was treated with DNase I (Invitrogen) and the RNA quality was checked in the UV spectrophotometer. For cDNA synthesis (1 μg/reaction) the RT^2^ first strand kit from SABiosciences was used. The resulting cDNA was diluted and qPCR was run as described in the PCR array protocol (SABiosciences RT^2^ Profiler PCR Array System) using a BioRad ICycler. Gene expression was tested using either Cancer Pathway Finder (untreated, IT 10 ng/ml) - or Tumor Metastasis (2 μM CsA, CsA + IT 10 ng/ml) array. There are primers for 84 test genes and 5 reference genes (B2M, HPRT1, RPL13A, GAPDH, and ACTB) on each 96-well plate. Data analysis was performed as described in the protocol from the manufacturer and by using their PCR Array Data Analysis Web portal (http://www.SABiosciences.com).

### Validation of PCR array data

Gene expression was validated in independent experiments with RNA isolated as described above. The high capacity RNA to DNA master mix was used for cDNA synthesis (1 μg RNA/ reaction). Gene expression was measured using qPCR analyses with TaqMan probes using the 7500 Real Time PCR machine (Applied Biosystems). Each sample was tested in duplicate. Fold change in expression was calculated using the comparative C_t_ method with RPL37A as a reference gene since the expression of this gene was similar in control and experimental groups. The gene list and corresponding probes are shown in Additional file 1: Table S1.

### Subcellular fractionation, gel electrophoresis, and antigen detection

Cells were grown to 70-80% confluency in 75 cm^2^ flasks and treated with MOC31PE and/or CsA for 24 h. The cells were washed with cold PBS and lysed in 500 μl SF buffer (250 mM sucrose, 20 mM Hepes, 10 mM KCl, 1.5 mM MgCl_2_, 1 mM EDTA and 1 mM EGTA, pH 7.5) and the protease inhibitor cocktail was added (MiniComplete, Roche). Cells were scraped from the flasks and the lysates were passed through 25G needles 10 times, and incubated on ice for 20 min. The nuclear pellet was centrifuged out at 720 g for 5 min and the resulting supernatant centrifuged at 10000 g for 10 min to separate the cytosolic (supernatant) and mitochondrial (pellet) fractions. Pellets were washed with 500 μl SF buffer, passed through 25G needles 10 times and re-centrifuged. Finally, the pellets were resuspended in 50 μl lysis buffer (10 mM Tris pH 7.5, 1% SDS, 1 mM Na_3_VO_4_, 0.1% Triton X-100, and 10% glycerol) and briefly sonicated. For total cell lysates, cells were lysed in boiling lysis buffer as previously described [17]. Proteins were resolved on 4-12% Nu-PAGE gels and blotted onto PVDF membranes for antigen detection. The purity of the fractions was validated with antibodies detecting α-tubulin (cytosol, Cell Signaling), lamin B1 (nucleus, Abcam), and F_1_F_0_-ATP synthase (mitochondria, Calbiochem). NR4A3 in the fractions was detected on separate blots using a polyclonal anti-NR4A3/NOR-1 antibody (Novus Biologicals). Chemiluminescence signals were recorded using the G:Box system with a CCD camera from SynGene and quantified using the provided GeneTool software.

### Statistical analyses

Statistical significance was evaluated with two-tailed Students t-test except for qPCR validations where non-parametric Mann–Whitney tests were used. In both tests p-values at 0.05 were considered statistically significant.

## Results

### MOC31PE immunotoxin inhibits protein synthesis and reduces cell viability

**Figure 1:**
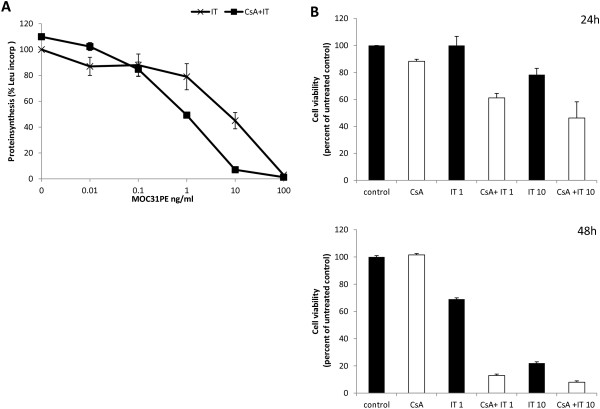
**The inhibitory effect of MOC31PE immunotoxin on protein synthesis and cell viability.** B76 cells were seeded in 48-well plates **(A)** and the next day the medium were changed and added IT and/or CsA. Protein synthesis was analysed after 24 h, measuring the amount of [^3^H]-leucine protein incorporation. Values for treated cells are shown as the percentage of the values obtained in non-treated control cells. One representative experiment with three wells (average ± SD) for each treatment is shown. The experiment was repeated twice. For the cell viability test **(B)**, cells were seeded in 96-well plates and added IT and/or CsA as described above. The MTS reagent was added after 24 h treatment and absorbance read after additional 4 h. Average ± SD of three independent experiments is shown. In each experiment each treatment were tested at least in triplicate wells. The lower graph **(B)** is one representative experiment of three independent experiments with treatment for 48 h. Average ± SD for each treatment tested in triplicate is shown.

The ovarian cancer cell line B76 was used to investigate intracellular effects of MOC31PE (IT) and CsA on protein synthesis and cell viability. The expression of EpCAM is high in these cells (as assessed by immunomagnetic selection with MOC31 antibody-coated beads; Additional file 2: Figure S1). The ID_50_ value for inhibition of protein synthesis was 8 ng/ml of MOC31PE (Figure 1A). Cell viability was quantified in a MTS assay. In 10 ng/ml IT-treated cells the viability was decreased to 80 percent of untreated control (Figure 1B). Protein synthesis was inhibited more efficiently when using the combination of IT with 2 μM CsA compared to IT treatment alone (Figure 1A). By combining IT with CsA the ID_50_ value for inhibition of protein synthesis with IT was ten times less than for IT alone. CsA alone showed none or negligible effects on protein synthesis and cytotoxicity. Although 1 ng/ml IT resulted in 20 percent reduction of protein synthesis, no significant reduction of cell viability was observed after 24 h (Figure 1B). By extending the incubation period to 48 h, the fraction of metabolically active cells decreased further in all treatment groups (Figure 1B). With 10 ng/ml IT alone 22 percent cell viability was observed, whereas the addition of CsA reduced the cell survival to only 13 percent.

### MOC31PE immunotoxin induces cell membrane damage and reduces cell migration

**Figure 2:**
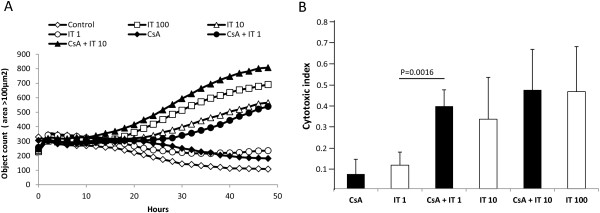
**Increased membrane leakage followed in an IncuCyte live-imaging device.** B76 cells were seeded in 96-well plates, IT and/or CsA were added and the fluorescence measured every second hour for 48 h **(A)**. Membrane leakage **(A)** was quantified using the built in fluorescent object count metric after adding the dsDNA-binding YoYo-1 together with IT and/or CsA (n = 6 wells). Filled markers are used for CsA (diamond) and combinations of CsA and IT (triangle, CsA + IT 10 ng/ml and circle, CsA + IT 1 ng/ml). Open markers for control (diamond) and IT 100 ng/ml (square), 10 ng/ml (triangle), 1 ng/ml (circle). Results were filtered and fluorescent objects with an area > 100 μm^2^ is shown. The cytotoxic index **(B)** is the ratio of fluorescent objects before and after lysis of the cells by adding Triton X-100. The average with SD of 4 independent experiments each with six wells for each treatment is shown. The CI index obtained for untreated cells was subtracted in each experiment.

Membrane damage was determined by quantifying the number of fluorescent objects in an IncuCyte, where cells were analyzed every second hour for up to 48 h after adding the fluorescent probe YoYo-1. Addition of YoYo-1 alone did not induce membrane damage. No differences in the number of fluorescent objects were observed during the first 12 h of treatment, indicating intact cell membranes. The fluorescence increased in IT treated cells after approximately 15 h (Figure 2A). Figure 2B shows the cytotoxic index (CI) obtained after 48 h treatment. A dose dependent IT-response was observed with doses from 1 ng/ml to 100 ng/ml. The membranes of the cells were more damaged by the combination of IT and CsA, decreasing the IT dose needed by a factor of approximately ten compared to IT alone. Only a minor increase in CI was seen after exposure to CsA alone.

**Figure 3:**
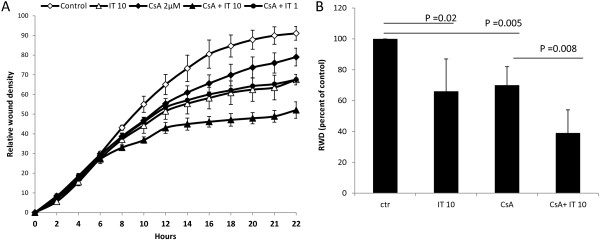
**Inhibitory effect of IT +/- CsA in a scratch-wound healing assay followed in an IncuCyte.** B76 cells were seeded in 96-well plates (Essen image lock) and scratch wounds made simultaneously in all wells using the wound maker tool. Relative wound density (RWD), defined as the ratio of the cell density in the wound over the cell density outside the wound, was measured every second hour for up to 22 h **(A)**. Open markers are for control (diamond) and IT 10 ng/ml (triangle) and filled markers are for CsA (diamond) and the combination of CsA and IT 10 ng/ml (triangle) or IT 1 ng/ml (circle). Average RWD after 22 h in five independent experiments is summarized in **(B)**. The value for the control wells in each experiment is taken as 100% and used for normalization. Statistical significance was calculated using the T-test (2-tails, unequal variance).

The wound healing assay mimics parts of the cancer metastasis process by measuring in vitro cell migration [18]. In control wells (untreated cells) the relative wound density (RWD) was 91 percent at start of the experiment (average of three wells, Figure 3A) and pictures taken after 22 h revealed almost complete wound closure (Additional file 3: Figure S2). In wells containing cells treated with IT (10 ng/ml), cell migration was inhibited as the RWD decreased to 66 percent (p = 0.02), and for CsA alone the RWD was 70 percent (p = 0.005). A further reduction was observed when cells were treated with a combination of IT and CsA (RWD = 39 percent, p = 0.008). Results from five independent experiments are summarized in Figure 3B.

### Effects of MOC31PE immunotoxin on gene expression

Previously, microarray analyses have revealed IT-induced differential expression of many transcripts [19]. To focus here on IT-induced changes in gene regulation two different PCR arrays were selected. One aim was to identify which cancer pathways were affected by IT treatment. The tumor metastasis array was used to study effects of the combination of CsA and IT, as this combination was previously shown to increase survival in a metastasis model in nude rats [8]. In two independent experiments, mRNA was isolated from cells treated for 24 h with 10 ng/ml IT. Expression of 13 genes was more than two-fold changed in IT treated samples compared to non-treated controls (Table 1). Increased gene expression was detected for 11 targets and decreased expression for two targets. The Cq values in the control samples were 25 or more cycles for nine of the 13 affected gene products. Six of the detected gene products belong to the angiogenesis pathway. Moreover, increased mRNA levels were found for the transcription factors Jun, ETS2, and NFκβ1, which e.g. regulate the expression of tumor angiogenesis genes. The highest increase in expression was observed for THBS1 (Thrombospondin, 5.8 fold increase) and PDGFβ (platelet derived growth factor, 9 fold increase). These genes were selected for validation using qPCR with Taqman probes. RNA was isolated in a set of independent experiments from IT-treated samples and from non-treated controls. In six experiments median fold changed expression for IT treated samples compared to non-treated controls was 5.4 for PDGFβ (ranging from 2.1 to 31.1, p < 0.02) and 10.5 for THBS1 (4.6 to 34.9, p < 0.02). The fold change values for the specific mRNA transcript varied between experiments most likely due to high Cq values i.e. low expression of the mRNA. Within each experiment the variation between technical replicates was low, typically less than 0.5 cycles.

**Table 1:** Fold change in gene expression comparing control (untreated cells) and 10 ng/ml IT treated B76 cells

**Cancer pathway**	**Gene**	**Description**	**Fold change**
Adhesion	ITGα3	Integrin α3	-2.1
Angiogenesis	FGFR2	Fibroblast growth factor receptor 2	2.1
	IFNβ1	Interferon β	3.8
	TNF	Tumor necrosis factor	3.8
	IL8	Interleukin 8	5.0
	THBS1	Thrombospondin	5.8
	PDGFβ	Platelet derived growth factor β	9.0
Cell cycle control and DNA damage repair	CDC25A	CDC25 phosphatase family	2.4
	CDKN1A	Cyclin dependent kinase inhibitor	2.4
Signal transduction molecules and transcription factors	ERBB2	Epidermal growth factor receptor family	-2.9
	ETS2	Transcription factor	3.2
	NFκβ1	Transcription factor	3.2
	JUN	Transcription factor	5.4

Results from two independent experiments are analysed together.

Using qPCR, possible effects of CsA alone and in combination with IT on expression of THBS1 and PDGFβ were also investigated. In CsA treated cells the expression of THBS1 and PDGFβ was two-fold reduced (n = 2) compared to the expression in untreated control cells. In four independent experiments, the combination treatment compared to CsA alone treatment gave median fold changed expression of 34.5 (from 4.4 to 76.3, p < 0.05) for THBS1 and of 13.9 for PDGFβ (4.5 to 41.3, p < 0.05).

In the Tumor Metastasis Array, 23 of 84 gene products (Table 2) were found to be at least two-fold differentially expressed in the combination treatment compared to CsA alone treatment. Only one mRNA, coding for MYCL-1, was down regulated. The Cq-values for 16 of 23 mRNAs were 25 or higher in CsA-treated cells. Four gene products, coding for NR4A3 (nuclear receptor family 4 member 3), KISS1 (kisspeptin 1), NME4 (expressed in non-metastatic cells 4), and MMP9 (matrix metalloproteinase 9) were selected for validation using qPCR, and the results from the PCR array experiments were confirmed. The median fold changed expression was 16.4 for NR4A3 (ranging from 3 to 25.4, p < 0.05), and 11.6 for KISS1 (ranging from 3 to 38.5, p < 0.05) in four independent experiments. NME4 was up-regulated 3.8 fold and MMP9 only weakly up-regulated (2.6 times). KISS1 and NR4A3 expression were increased also in cells treated with IT alone, confirming that the differential gene expression was independent of CsA. Expression of these transcripts was also analyzed after IT treatment of the ovarian cancer cell line HOC7. The inhibitory effect of MOC31PE on protein synthesis and decreased cell viability in HOC7 cells is shown in the Additional file 4: Figure S3 and Additional file 5: Figure S4. The IT induced increase of NR4A3 expression was confirmed in this cell line (Additional file 6: Figure S5) and two-fold increase of THBS1, PDGFβ, and KISS1 transcripts were also detected.

**Table 2:** Fold change in gene expression comparing CsA treated B76 cells with or without 10 ng/ml IT

**Cancer pathway**	**Gene**	**Description**	**Fold change**
Cell adhesion	PNN	Pinin, desmosome-associated protein	2.2
	FAT1	Cadherin-related tumor suppressor homolog	2.3
Cell cyclus	BRMS1	Breast cancer metastase suppressor, transcriptional repressor	2.0
	NF2	Neurofibromin	2.2
Cell cyclus and transcription factor	RB1	Retinoblastoma, tumor suppressor, transcriptional repressor	2.2
	TP53	Tumor suppressor, transcription factor	2.3
Cell cyclus or cell proliferation	NME1	Expressed in Non-Metastatic cells, nucleoside diphosphate kinase	3.2
Cell growth and proliferation	SSTR2	Somatostatin receptor 2, ligand somatostatin 14/28	2,1
	DENR	Density-regulated protein, involved in translation	2.6
	KISS1R	Receptor for KISS1	2.8
	FLT4	Receptor tyrosine kinase, ligand VEGF C/D	3.1
	CXCR4	CXC chemokine receptor, ligand SDF-1	3.4
	EPHB2	Receptor tyrosine kinase, ligand ephrin-family members	10.3
Invasion	MMP10	Matrix metalloproteinase	2.4
	MMP9	Matrix metalloproteinase	5.3
Other	METAP2	Methionyl aminopeptidase	2.1
	CD82	Metastasis suppressor	2.2
	CTSK	Cathepsin K, cysteine protease	4.2
	NME4	Expressed in Non-Metastatic cells, nucleoside diphosphate kinase	7.6
	KISS1	Metastasis suppressor	14.6
Transcription factor	MYCL1	Myc-related	-3.8
	SMAD4	SMAD family member,	2.0
	NR4A3	Nuclear-receptor subfamily 4 member A3, potential transcriptional activator	20.6

Results from two independent experiments are analysed together.

### Effects of MOC31PE immunotoxin on NR4A3 protein expression and subcellular localization

**Figure 4:**
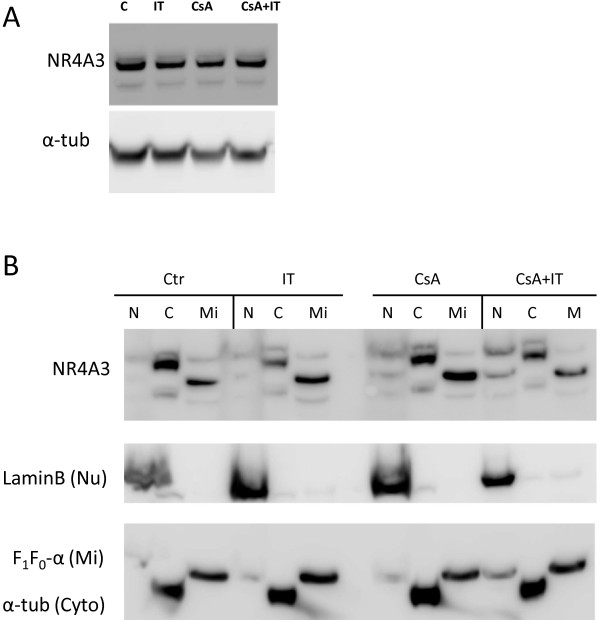
**Mechanisms for NR4A3 protein involvement during IT-induced cell death.** B76 cells were seeded in 25 cm^2^ flasks and treated for 24 h with IT (10 ng/ml), CsA (2 μM) or the combination when cells were 70% confluent. Cell lysates were prepared and 15 μg total protein added each SDS-PAGE lane **(A)**. The apparent molecular weight for the main band is 60 kDa and around 55 kDa for the lower band. In **(B)** cells were seeded in 75 cm^2^ flasks and treated as in **(A)**. Adherent cells were used for subcellular fractionation. Immunoblots were probed with the NR4A3 antibody or subcellular fraction marker antibodies as indicated. The immunoblots are from one representative experiment of three independent experiments.

In the tested ovarian cancer cell lines, B76 and HOC7, treated with IT the largest increase in mRNA expression was observed for NR4A3. Immunoblot of B76 protein lysates with anti-NR4A3 antibody gave two proteins bands with apparent molecular weight of 55 and 60 kDa. No significant differences in protein level were seen when comparing the different treatments (Figure 4A). Pro-survival as well as pro-apoptotic functions have been ascribed to NR4-family members. The pro-survival effect is due to transcription factor activity and localization in the nucleus, whereas the pro-apoptotic effect has been suggested to require mitochondrial localization. We performed subcellular fractionation to identify the subcellular localization and possibly infer from this the mechanism for NR4A3 involvement during IT treatment. Three fractions enriched in either nuclear proteins, cytosolic proteins, or mitochondrial proteins were obtained and the purity of the different fractions was validated by immunoblotting (Figure 4B). The cytosolic and nuclear fractions were acceptably pure (less than 10% contamination), whereas the mitochondrial marker protein was detected also in the nuclear fraction, especially when the cells had been treated with IT in combination with CsA. In this case the nuclear fraction contained 24 percent of the total F_1_F_0_-α ATP-synthase compared to 4 percent in the corresponding fraction from control cells. In fractions from control cells NR4A3 was detected mainly as a 60 kDa band in the cytosol and as a 55 kDa band in the mitochondrial fraction. A faint band around 60 kDa was detected in the nuclear fraction, but in this fraction a 100 kDa band was also observed (not shown). IT treatment increased the amount of NR4A3in the mitochondrial fraction, indicating a pro-apoptotic function of NR4A3 (Figure 4B). This increase was also observed in mitochondrial fraction from CsA treated cells, and was accompanied by increased NR4A3 in the nuclear fraction, suggesting increased transcription of NR4A3 regulated genes. NR4A3 was reduced in the mitochondrial fraction from IT + CsA treated cells and further increased in the nuclear fraction. Increased amount of the two NR4A3 bands and detection of the mitochondrial marker in the nuclear fraction indicates altered intracellular compartment for mitochondrial proteins as could be expected in cells undergoing apoptosis.

## Discussion

The major limitation to curative therapy for ovarian cancer is acquired drug resistance to the chemotherapeutic agents used, such as Carboplatin and Paclitaxel. An additional drawback is the induced severe side-effects, mainly caused by the non-cancer cell specificity of the agents, reducing the patients’ quality of life. It is therefore necessary to identify novel drugs, which circumvent these disadvantages for successful treatment of ovarian cancer. In the present study, we have demonstrated in several different assays that the MOC31PE effectively inhibits protein synthesis, proliferation and cell survival of ovarian cancer cells, B76 and HOC7. Previously, we have reported in other tumor types synergistic cytotoxic effects of combining MOC31PE and CsA *in vitro* and in an experimental metastasis model in animals [8]. In agreement with previous results in other tumor types, these effects are potentiated when cells are simultaneously exposed to the immunosuppressant CsA.

The MOC31PE only binds to and kill cells expressing the antigen EpCAM, which is expressed in more than 90% of all epithelial ovarian carcinomas and to a negligible amount on normal cells, reducing the possibility of IT induced side effects in patients. In a recently conducted Phase I clinical study with MOC31PE, the tolerable profile was satisfactory (Andersson et al., in preparation), which is encouraging for clinical evaluation of MOC31PE against ovarian carcinoma. Interestingly, Phase I and II trials with CsA have shown beneficial effects on chemoresistance in patients with ovarian cancer [11,20] indicating that the combination of MOC31PE and CsA could be used for recurrent ovarian cancer.

Gene expression analysis was performed to identify affected signaling pathways induced by the treatments and several interesting candidate genes were found. In the Cancer Pathway Finder array, the majority of the genes affected by MOC31PE were related to angiogenesis, reflecting the importance of this cancer pathway in B76 cell growth. The two genes with the highest fold increase in expression on the array, PDGFβ and THBS1, was confirmed by qPCR. The PDGF network was recently identified as a biomarker for prognosis in ovarian cancer where higher levels of PDGF pathway activity were associated with reduced survival [21]. The angiogenesis inhibitor Bevacizumab (Avastin), that binds to VEGF A, is an used molecular target agent in ovarian cancer [22]. Given the importance of the PDGF pathway, targeting of VEGF, PDGF, and FGF at the same time may be more effective than targeting only VEGF [23]. THBS1 was the first endogenous angiogenesis inhibitor identified [24]. A role in cancer progression and cancer inhibition has been ascribed to the protein, and different effects of THBS1 depending on the phase of the progression have been suggested [25]. In an early stage, high stromal expression of THBS1 inhibits tumor growth whereas later in the vascularized tumor THBS1 may increase the adhesive properties of tumor cells or modulate extracellular matrix proteins thereby promoting tumor invasion. We observed that CsA mono-treatment inhibited migration and reduced expression of some transcripts, including THBS1 in addition to potentiating IT effects. Calcineurin, the phosphatase inhibited by CsA, has been reported to regulate transcription of CTSK [26] and CXCR4 [27]; two of five other affected genes. The inhibition of B76 cell migration by IT + CsA treatment may be a result of reduced THBS1 and/or MMP9 protein levels since increased transcription cannot be accompanied by increased translation due to IT-induced protein synthesis inhibition. In the tumor metastasis array mainly increased gene expression was seen when comparing CsA alone versus CsA + MOC31PE treatment of B76 cells. Examples of genes influenced are the metastasis suppressor KISS1 and its receptor. In ovarian carcinoma the increased expression of KISS1 has been shown to inhibit cell migration [28]. This might support the results from the scratch-wound healing assay showing decreased migration in the B76 cells treated with MOC31PE alone or MOC31PE + CsA. Higher expression of KISS1 may also sensitize cancer cells for chemotherapy [29]. Thus our results might support a contribution of MOC31PE as a supplement also to reduce chemotherapy resistance in ovarian cancer treatment.

The largest up-regulation was observed for the nuclear hormone receptor NR4A3, a member of the NR4A subfamily with poorly understood biological function and unknown physiological ligands [30]. Depending on the context, NR4A transcription factors may be pro-survival factors or induce cell death [31]. Knock-out mice without NR4A3 (Nor-1) and NR4A1 (Nur77) developed spontaneous acute myeloid leukemia [32] suggesting tumor suppressing effects. In cancer cells, growth factors and mitogens induce expression of these transcription factors suggesting a role in cancer growth [31]. However, induction of NR4A1 also occurs in response to apoptosis inducing factors in cancer cells. When translocated to mitochondria NR4A1 binds BCL-2, thereby inducing apoptotic cell death [31] and during apoptosis in thymocytes mitochondrial targeting of NR4A3 was observed [33]. In B76 cells, the majority of the NR4A3 protein was located in the cytosol. Two main changes in intracellular distribution were observed. MOC31PE or CsA shifted the protein to the mitochondrial fraction compatible with induction of apoptosis. Especially in MOC31PE + CsA treated cells increased NR4A3 was detected in the nuclear fraction. Increased amount of 60 kDa protein points to increased transcription of its target genes. Since increased 55 kDa protein in the nuclear fraction was accompanied by increased mitochondrial marker protein, and the nuclear fraction was pelleted at low speed, this implies that the mitochondrial mass has increased or that mitochondria have fused to larger structures. This is most likely an effect of the ongoing cell death. The increase in NR4A3 transcript, signals a need for NR4A3 protein synthesis. No corresponding increased NR4A3 protein was detected as IT inhibits protein synthesis, but translocation of NR4A3 to mitochondria enriched fractions suggests a role for this protein in MOC31PE-induced cell-death.

In summary, these results show that a PE-containing IT, MOC31PE, induces transcription of mRNAs for genes involved in angiogenesis and tumor metastasis. In addition, the therapeutic use of MOC31PE alone or in combination with CsA may provide an approach to the treatment of recurrent/chemoresistant ovarian carcinoma, but further investigation is needed to elucidate the effect of MOC31PE and CsA in ovarian cancer models *in vivo*.

## Competing interests

The authors declare that they have no competing financial or non-financial competing interests.

## Authors’ contributions

MTW provided, analyzed, and interpreted experimental data and drafted the manuscript. HB was involved in experimental data acquisition, ØF and KF participated in interpretation of the data and revisions of the manuscript, YA substantially contributed to the conception and design of the study, interpretation of experimental data and major revisions of the manuscript. All authors have approved the final version of the manuscript.

## Supplementary Material

Additional file 1: Table S1 Taqman probe/primers from Applied Biosystems (Life Technology) that were used for validation gene-expression data that were observed with the PCR array technology. Click here for file

Additional file 2: Figure S1 Cell surface expression of EpCAM was detected using magnetic beads coated with the MOC31 antibody. B76 cells were detached from the plastic with EDTA and incubated for 30 min with these beads or control beads (IgG). Upper panel show very good binding and thus high expression of the antigen EpCAM whereas no binding was seen with control beads. Click here for file

Additional file 3: Figure S2 Pictures of B76 cells taken immediately after scratching confluent cell layers (0 h) and after incubating wells with media containing MOC31PE (10 ng/ml) or CsA + MOC31PE for 24 hours in the scratch assay. Control wells were added only growth media. After 24 h the wound is closed in the control well and still open in treated wells. Click here for file

Additional file 4: Figure S3 Protein synthesis in HOC-7 ovarian cancer cells after 24 h incubation with MOC31PE. A dose-dependent decreased incorporation of ^3^H-leu was observed compared with the incorporation of ^3^H-leu in control cells. Click here for file

Additional file 5: Figure S4 Effect of MOC31PE on HOC-7 ovarian cancer cell viability measured using the MTS-assay. Cells were incubated with IT for 24 and 48 hours as indicated. Click here for file

Additional file 6: Figure S5 Gene expression of selected genes in HOC-7 ovarian cancer cells tested in qPCR with Taqman probes. RNA was isolated from untreated cells and cells treated with 10 ng/ml IT in 2–4 independent experiments. Fold-changed expression with standard deviation is shown. The Cq in control samples were higher than 25. Click here for file

## References

[B1] Ozols RF Bundy BN Greer BE Fowler JM Clarke-Pearson D Burger RA Phase III trial of carboplatin and paclitaxel compared With cisplatin and paclitaxel in patients with optimally resected stage III ovarian cancer: a gynecologic oncology group study J Clin Oncol 2003 21 3194 3200 10.1200/JCO.2003.02.153 12860964

[B2] Bamias A Pignata S Pujade-Lauraine E Angiogenesis: a promising therapeutic target for ovarian cancer Crit Rev Oncol/Hematol 2012 84 314 326 10.1016/j.critrevonc.2012.04.002 22575381

[B3] Patriarca C Macchi RM Marschner AK Mellstedt H Epithelial cell adhesion molecule expression (CD326) in cancer: a short review Cancer Treat Rev 2011 38 68 75 21576002 10.1016/j.ctrv.2011.04.002

[B4] Tveito S Andersen K Karesen R Fodstad O Analysis of EpCAM positive cells isolated from sentinel lymph nodes of breast cancer patients identifies subpopulations of cells with distinct transcription profiles Breast Cancer Res 2011 13 R75 10.1186/bcr2922 21816090 PMC3236339

[B5] Köbel M Kalloger SE Boyd N McKinney S Mehl E Palmer C Ovarian carcinoma subtypes are different diseases: implications for biomarker studies PLoS Med 2008 5 1749 1759 10.1371/journal.pmed.0050232 PMC2592352 19053170

[B6] Emmanuel C Gava N Kennedy C Balleine RL Sharma R Wain G Comparison of expression profiles in ovarian epithelium *In Vivo* and ovarian cancer identifies novel candidate genes involved in disease pathogenesis PLoS ONE 2011 6 e17617 10.1371/journal.pone.0017617 21423607 PMC3057977

[B7] Risberg K Fodstad Ø Andersson Y Anti-melanoma activity of the 9.2.27PE immunotoxin in dacarbazine resistant cells J Immunother 2010 33 272 278 10.1097/CJI.0b013e3181c54991 20445347

[B8] Andersson Y Engebraaten O Fodstad O Synergistic anti-cancer effects of immunotoxin and cyclosporin in vitro and in vivo Br J Cancer 2009 101 1307 1315 10.1038/sj.bjc.6605312 19773757 PMC2768448

[B9] Xavier M Drug immunosuppression therapy for adult heart transplantation. Part 1: immune response to allograft and mechanism of action of immunosuppressants Ann Thorac Surg 2004 77 354 362 10.1016/j.athoracsur.2003.07.006 14726104

[B10] Obchoei S Wongkhan S Wongkham C Li M Yao Q Chen C Cyclophilin A: potential functions and therapeuti target for human cancer Med Sci Monit 2009 15 RA221 RA232 19865066

[B11] Morgan RJJ Synold T Gandara D Muggia F Scudder S Reed E Phase II trial of carboplatin and infusional cyclosporine with alpha-interferon in recurrent ovarian cancer: a California cancer consortium trial Int J Gynecol Cancer 2007 17 373 378 10.1111/j.1525-1438.2007.00787.x 17362315

[B12] Sood A Sorosky J Squatrito R Skilling J Anderson B Buller R Cyclosporin A reverses chemoresistance in patients with gynecologic malignancies Neoplasia 1999 1 118 122 10.1038/sj.neo.7900019 10933045 PMC1508129

[B13] Marth C Zeimet AG Herold M Brumm C Windbichler G Müller-Holzner E Different effects of interferons, interleukin-1β and tumor necrosis factor-α in normal (OSE) and malignant human ovarian epithelial cells Int J Cancer 1996 67 826 830 10.1002/(SICI)1097-0215(19960917)67:6<826::AID-IJC12>3.0.CO;2-# 8824555

[B14] Buick RN Pullano R Trent JM Comparative properties of five human ovarian adenocarcinoma cell lines Cancer Res 1985 45 3668 3676 4016745

[B15] Myklebust AT Beiske K Pharo A Davies CL Aamdahl S Fodstad Ø Selection of anti-SCLC antibodies for diagnosis of bone marrow metastasis Br J Cancer 1991 63 49 53 PMC2204098 1645572

[B16] Engebraaten O Sivam G Juell S Fodstad Ø Systemic immunotoxin treatment inhibits formation of human breast cancer metastasis and tumor growth in nude rats Int J Cancer 2000 88 970 976 10.1002/1097-0215(20001215)88:6<970::AID-IJC21>3.0.CO;2-Q 11093823

[B17] Andersson Y Le H Juell S Fodstad Ø AMP-activated protein kinase protects against ant-epidermal growth factor recetor-pseudomonas exotoxin A immunotoxin-induced MA11breast cancer cell death Mol Cancer Res 2006 5 1050 1059 10.1158/1535-7163.MCT-05-0318 16648577

[B18] Arwert EN Hoste E Watt FM Epithelial stem cells, wound healing and cancer Nat Rev Cancer 2012 12 170 180 10.1038/nrc3217 22362215

[B19] Risberg K Guldvik IJ Palchaudhuri R Xi Y Fodstad Ø Hergenrother PJ Triphenylmethyl derivatived enhances the anticancer effect of immunotoxins J Immunother 2011 34 438 447 10.1097/CJI.0b013e31821e00ae 21577141

[B20] Chambers S Chambers J Davis C Kohorn E Schwartz P Lorber M Pharmacokinetic and phase I trial of intraperitoneal carboplatin and cyclosporine in refractory ovarian cancer patients J Clin Oncol 1997 15 1945 1952 9164206 10.1200/JCO.1997.15.5.1945

[B21] Ben-Hamo R Efroni S Biomarker robustness reveals the PDGF network as driving disease outcome in ovarian cancer patients in multiple studies BMC Syst Biol 2012 6 3 10.1186/1752-0509-6-3 22236809 PMC3298526

[B22] Itamochi H Targeted therapies in epithelial ovarian cancer: molecular mechanism of action World J Biol Chem 2010 1 209 2200 10.4331/wjbc.v1.i7.209 21537476 PMC3083967

[B23] Bell-McGuinn K Konner J Tew W Spriggs DR New drugs for ovarian cancer Ann Oncol 2011 22 viii77 viii82 22180408 10.1093/annonc/mdr531

[B24] Good DJ Polverini PJ Rastinejad F Le Beau MM Lemons RS Frazier WA A tumor suppressor-dependent inhibitor of angiogenesis is immunologically and functionally indistinguishable from a fragment of thrombospondin Proc Natl Acad Sci 1990 87 6624 6628 10.1073/pnas.87.17.6624 1697685 PMC54589

[B25] Kazerounian S Yee KO Lawler J Thrombospondins in cancer Cell Mol Life Sci 2008 65 700 712 10.1007/s00018-007-7486-z 18193162 PMC2752021

[B26] Combs MD Braitsch CM Lange AW James JF Yutzey KE NFATC1 promotes epicardium-derived cell invasion into myocardium Dev 2011 138 1747 1757 10.1242/dev.060996 PMC3074451 21447555

[B27] Cristillo AD Bierer BE Regulation of CXCR4 expression in human T lymphocytes by calcium and calcineurin Mol Immunol 2003 40 539 553 10.1016/S0161-5890(03)00169-X 14563373

[B28] Hata K Dhar DK Watanabe Y Nakai H Hoshiai H Expression of metastin and a G-protein-coupled receptor (AXOR12) in epithelial ovarian cancer Eur J Cancer 2007 43 1452 1459 10.1016/j.ejca.2007.03.004 17442564

[B29] Jiffar T Yilmaz T Lee J Hanna E El-Naggar A Yu D KiSS1 mediates platinum sensitivity and metastasis suppression in head and neck squamous cell carcinoma Oncogene 2011 30 3163 3173 10.1038/onc.2011.39 21383688 PMC3136629

[B30] Pearen MA Muscat GEO Minireview: nuclear hormone receptor 4A signaling: implications for metabolic disease Mol Endocrinol 2010 24 1891 1903 10.1210/me.2010-0015 20392876 PMC5417389

[B31] Moll U Marchenko M Zhang X p53 and Nurr77/TR3 - transcription factors that directly target mitochondria for cell death induction Oncogene 2006 25 4725 4743 10.1038/sj.onc.1209601 16892086

[B32] Mullican S Zhang S Konopleva M Ruvolo V Andreeff M Milbrandt J Abrogation of nuclear receptors Nr4a3 and Nr4a1 leads to development of acute myeloid leukemia Nat Med 2007 13 730 735 10.1038/nm1579 17515897

[B33] Thompson J Burger ML Whang H Winoto A Protein kinase C regulates mitochondrial targeting of Nur77 and its family member Nor-1 in thymocytes undergoing apoptosis Eur J Immunol 2010 40 2041 2049 10.1002/eji.200940231 20411565 PMC3076209

