# Supplementary material for: The MOC31PE immunotoxin reduces cell migration and induces gene expression and cell death in ovarian cancer cells
Source: J Ovarian Res. 2014 Feb 15;7:23. doi: 10.1186/1757-2215-7-23 (PMC3931919; doi:10.1186/1757-2215-7-23)
Supplement: Additional file 1: Table S1 — Taqman probe/primers from Applied Biosystems (Life Technology) that were used for validation gene-expression data that were observed with the PCR array technology. [file 1757-2215-7-23-S1.pdf]

Additional table 1: Taqman probes used for gene-expression validation

Gene	Taqman probe/primer (Applied)
THBS-1	Hs00962908_m1
PDGF β	Hs00966522_m1
KISS1	Hs00158486_m1
NR4A3	Hs00545007_m1
NME4	Hs00359037_m1
MMP9	Hs00234579_m1
RPL37a	Hs01102345_m1
