# Supplementary figures and images for: The MOC31PE immunotoxin reduces cell migration and induces gene expression and cell death in ovarian cancer cells
Source: J Ovarian Res. 2014 Feb 15;7:23. doi: 10.1186/1757-2215-7-23 (PMC3931919; doi:10.1186/1757-2215-7-23)

# Additional fig.1

MOC31

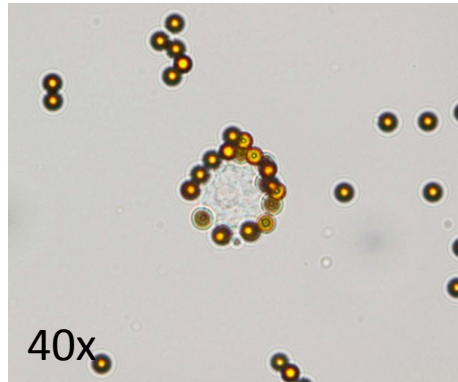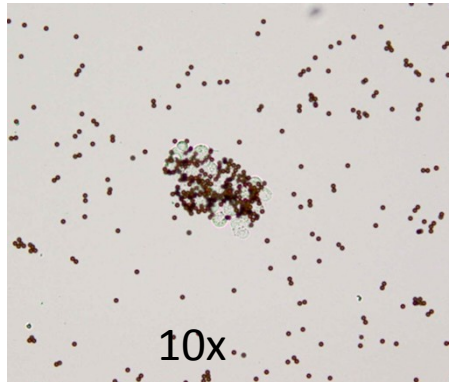

IgG

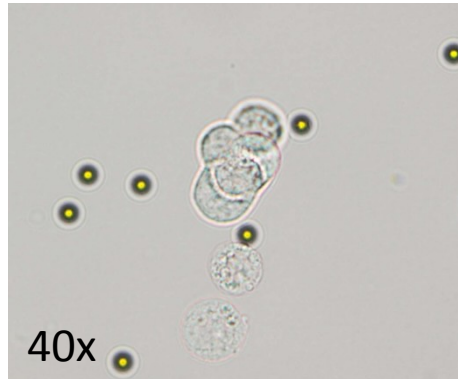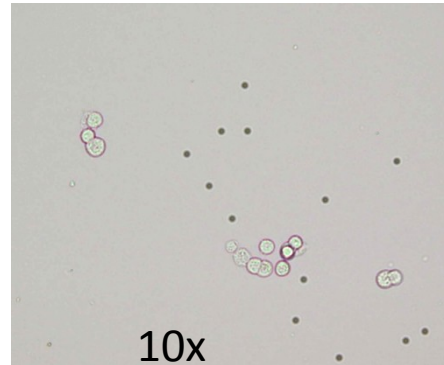

Supplement: Additional file 2: Figure S1 — Cell surface expression of EpCAM was detected using magnetic beads coated with the MOC31 antibody. B76 cells were detached from the plastic with EDTA and incubated for 30 min with these beads or control beads (IgG). Upper panel show very good binding and thus high expression of the antigen EpCAM whereas no binding was seen with control beads. [file 1757-2215-7-23-S2.pdf]

Additional fig.2 Scratch-wound assay

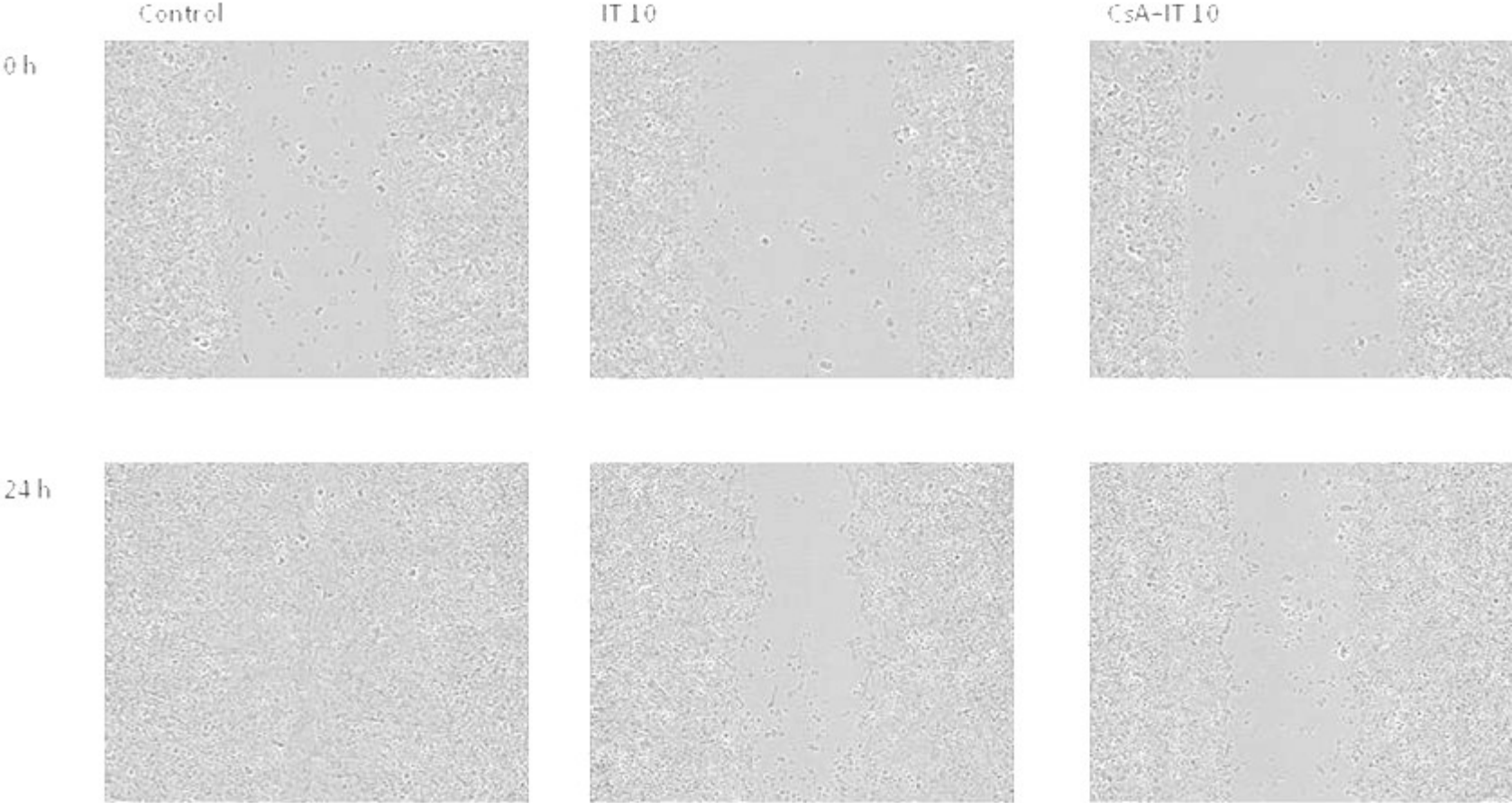

Supplement: Additional file 3: Figure S2 — Pictures of B76 cells taken immediately after scratching confluent cell layers (0 h) and after incubating wells with media containing MOC31PE (10 ng/ml) or CsA + MOC31PE for 24 hours in the scratch assay. Control wells were added only growth media. After 24 h the wound is closed in the control well and still open in treated wells. [file 1757-2215-7-23-S3.pdf]

Additional fig.3 protein synthesis in HOC-7 ovarian cancer cells

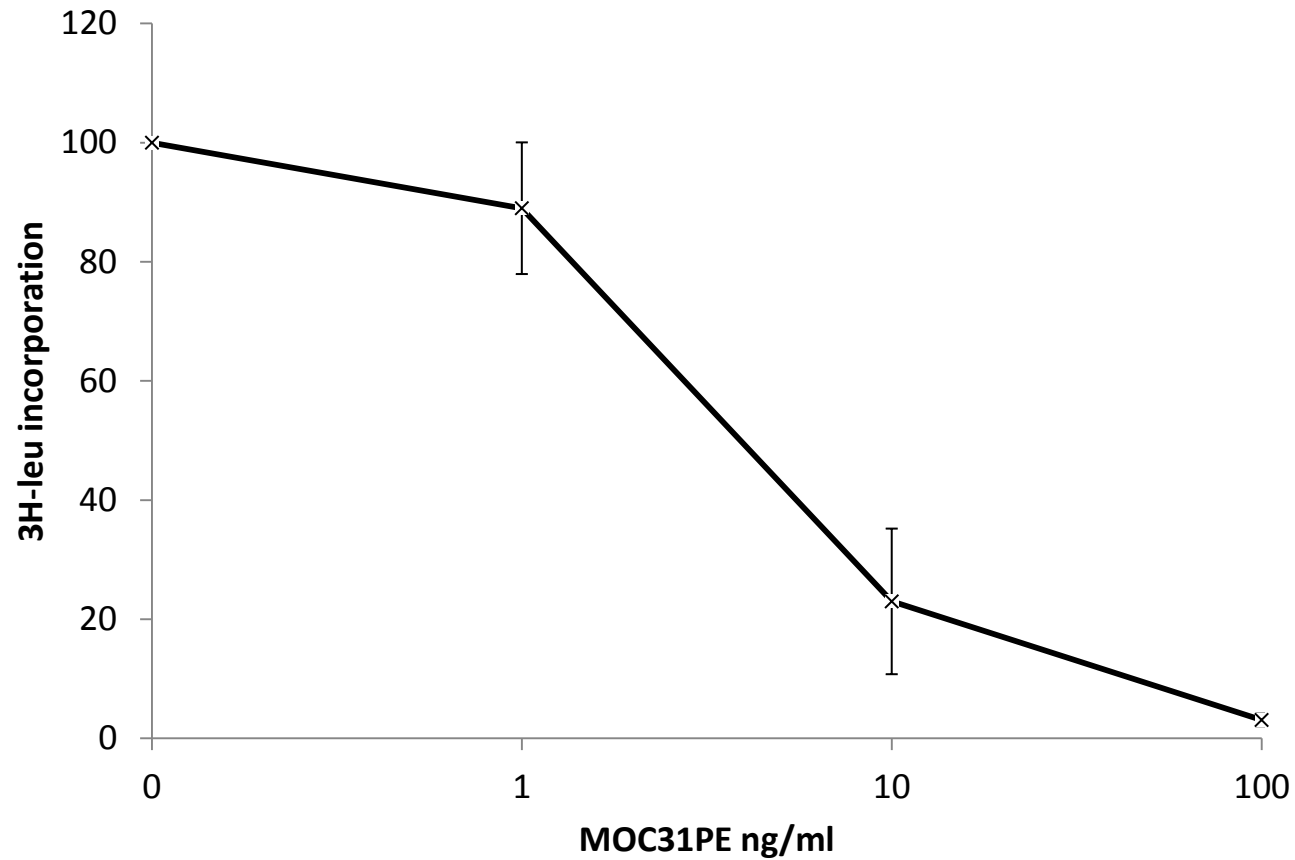

Supplement: Additional file 4: Figure S3 — Protein synthesis in HOC-7 ovarian cancer cells after 24 h incubation with MOC31PE. A dose-dependent decreased incorporation of 3H-leu was observed compared with the incorporation of 3H-leu in control cells. [file 1757-2215-7-23-S4.pdf]

Additional fig.4 cell viability in HOC-7 ovarian cancer cells

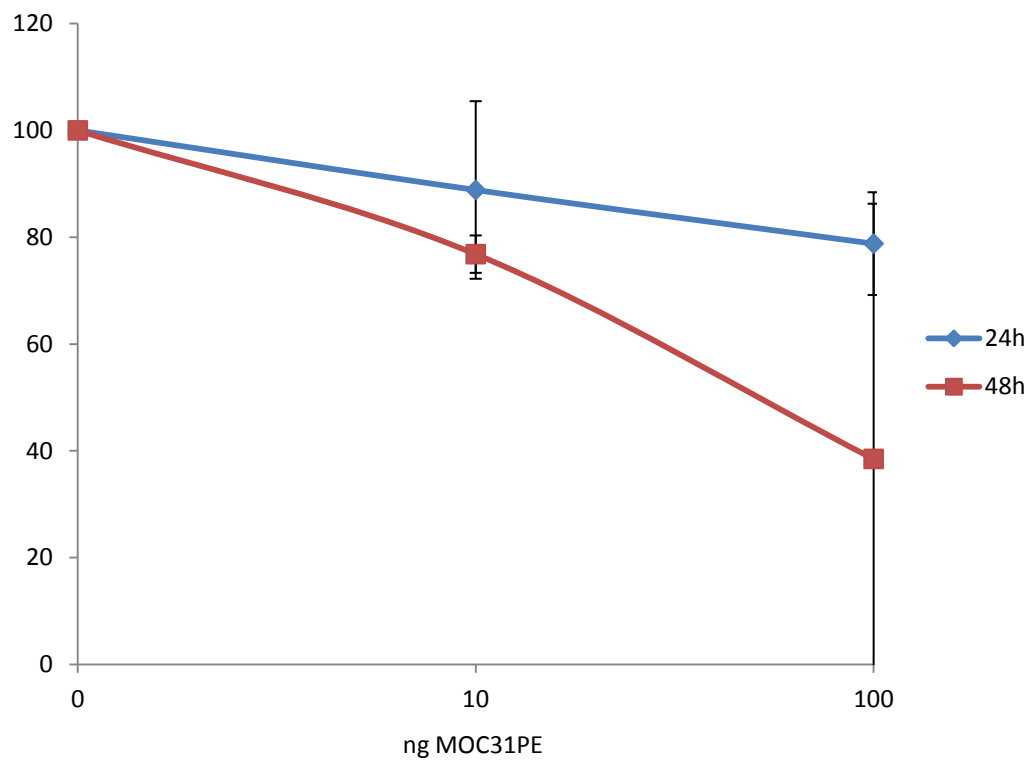

Supplement: Additional file 5: Figure S4 — Effect of MOC31PE on HOC-7 ovarian cancer cell viability measured using the MTS-assay. Cells were incubated with IT for 24 and 48 hours as indicated. [file 1757-2215-7-23-S5.pdf]

Additional fig.5: qPCR of selected genes in HOC-7 ovarian cancer cells

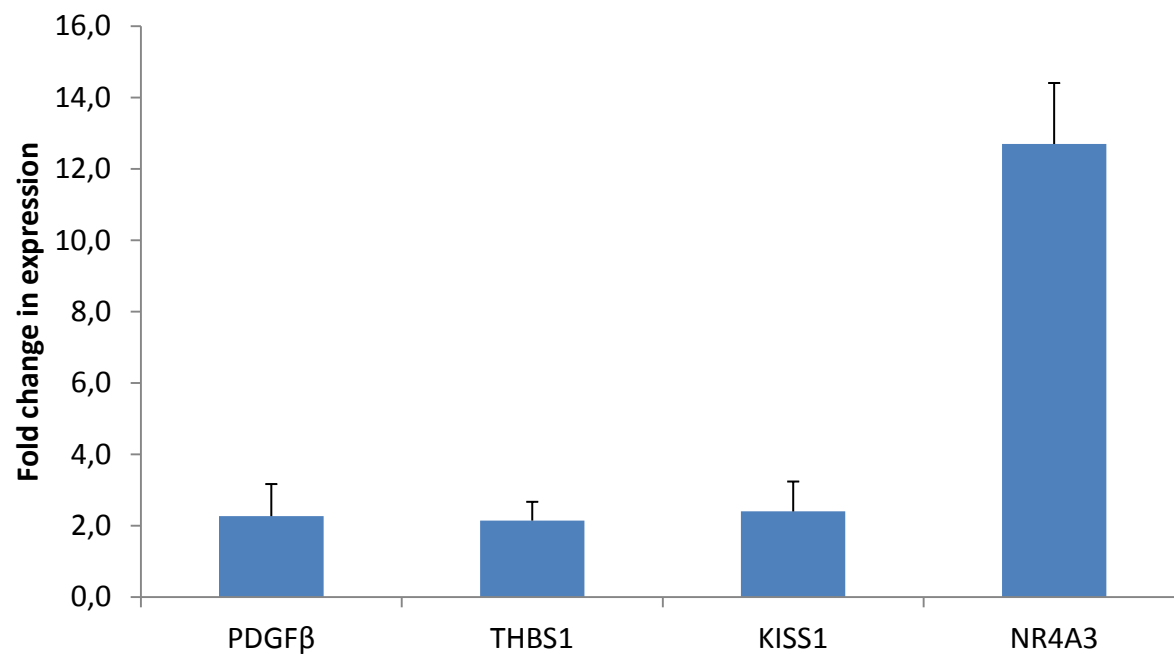

Supplement: Additional file 6: Figure S5 — Gene expression of selected genes in HOC-7 ovarian cancer cells tested in qPCR with Taqman probes. RNA was isolated from untreated cells and cells treated with 10 ng/ml IT in 2–4 independent experiments. Fold-changed expression with standard deviation is shown. The Cq in control samples were higher than 25. [file 1757-2215-7-23-S6.pdf]
